# Volumetric (3D) bladder dose parameters are more reproducible than point (2D) dose parameters in vaginal vault high-dose-rate brachytherapy

**DOI:** 10.1038/srep28074

**Published:** 2016-06-14

**Authors:** Lucas Gomes Sapienza, Adriana Flosi, Antonio Aiza, Antonio Cassio de Assis Pellizzon, Rubens Chojniak, Glauco Baiocchi

**Affiliations:** 1 Department of Radiation Oncology, A.C. Camargo Cancer Center, São Paulo, 01509-010, Brazil; 2 Department of Radiation Oncology, Clínicas Oncológicas Integradas (COI-RJ), Rio de Janeiro, 22793-080, Brazil; 3 Department of Radiology, A.C. Camargo Cancer Center, São Paulo, 01509-010, Brazil; 4 Department of Gynecologic Oncology, A.C. Camargo Cancer Center, São Paulo, 01509-010, Brazil

## Abstract

There is no consensus on the use of computed tomography in vaginal cuff brachytherapy (VCB) planning. The purpose of this study was to prospectively determine the reproducibility of point bladder dose parameters (D_ICRU_ and maximum dose), compared with volumetric-based parameters. Twenty-two patients who were treated with high-dose-rate (HDR) VCB underwent simulation by computed tomography (CT-scan) with a Foley catheter at standard tension (position A) and extra tension (position B). CT-scan determined the bladder ICRU dose point in both positions and compared the displacement and recorded dose. Volumetric parameters (D0.1cc, D1.0cc, D2.0cc, D4.0cc and D50%) and point dose parameters were compared. The average spatial shift in ICRU dose point in the vertical, longitudinal and lateral directions was 2.91 mm (range: 0.10–9.00), 12.04 mm (range: 4.50–24.50) and 2.65 mm (range: 0.60–8.80), respectively. The D_ICRU_ ratio for positions A and B was 1.64 (*p* < 0.001). Moreover, a decrease in Dmax was observed (*p* = 0.016). Tension level of the urinary catheter did not affect the volumetric parameters. Our data suggest that point parameters (D_ICRU_ and Dmax) are not reproducible and are not the ideal choice for dose reporting.

Postoperative vaginal cuff brachytherapy (VCB) with or without external beam radiation therapy (EBRT) is an integral component of the adjuvant treatment of endometrial cancer^1^, and this combination is an option in certain cases of cervical cancer^2^.

The documentation of the dose to organs at risk (OARs) during gynecological intra-cavitary brachytherapy was formally stated by the International Commission on Radiation Units and Measurements (ICRU) in 1985^3^. Published in 2006^4^, the Groupe Européen de Curiethérapie (GEC) and the European Society for Radiotherapy and Oncology (ESTRO) recommendations for 3D image-based brachytherapy, directed to cervical cancer, advocate documentation of the dose to the bladder with the ICRU reference point and D0.1cc, D1.0cc and D2.0cc (volumetric parameters). In 2012, the American Brachytherapy Society (ABS) published a consensus guideline on recommendations for VCB^5^, including reporting the dose to adjacent organs, particularly the bladder and rectum, but did not specify the exact parameters.

Currently, the choice between point dose parameters, such as ICRU bladder point dose (D_ICRU_) and maximum bladder dose (Dmax), and volumetric parameters relies solely on dosimetric evidence^6^,^7^,^8^,^9^,^10^,^11^,^12^. Several studies have compared ICRU and volumetric dose, but the lack of a relation or difference between them prevents either from being recommended over the other. Thus, evidence of the superiority of 3D planning in terms of predicting toxicity over 2D planning—or a technical limitation of the 2D planning—must be provided to justify the use of volumetric parameters in the post-hysterectomy setting.

Our aim was to prospectively demonstrate the limitations in reproducing conventional point-based dosimetry after comparing bladder volumetric dose parameters with bladder point dose parameters in the treatment of vaginal apex treatment, using two different tension levels for positioning of the Foley catheter.

## Results

Twenty-two patients were prospectively enrolled in this study between June 2014 and February 2015, totaling 44 CT scans (22 in position A and 22 in position B). Seventeen subjects had endometrial cancer (11 stage I, 1 stage II, 5 stage III), and 5 had cervical cancer (4 stage I and 1 stage II). Fifteen patients received EBRT + BT (53% tri-dimensional conformal radiation therapy and 47% intensity-modulated radiation therapy), and 7 received BT alone. The diameter of the cylinder was 26 mm in 36.4% of patients and 30 mm in 63.6%, according to patient anatomy. Two patients received para-aortic irradiation. There were no complications that were related to insertion of the Foley catheter at the time of the CT scan. The patients were treated with the catheter in standard position.

Data from all 44 treatment plans were available for analysis. None of the parameters (in terms of average doses) was normally distributed.

### Comparison between point dosimetry and volumetric dosimetry

Based on a normalized dose value (based on the percentage of the prescribed dose), the average D_ICRU_ documented in position A was 2.68 Gy, comprising 44.6% of the total prescribed dose.

Compared with standard tension, the bladder D_ICRU_ value differed significantly from Dmax (p < 0.0001), with a Dmax/D_ICRU_ ratio of 2.32. The same results were seen with the volumetric parameters and in comparison with position B (Table 1).

### Impact of Foley catheter tension on dose parameters

**Figure 1:**
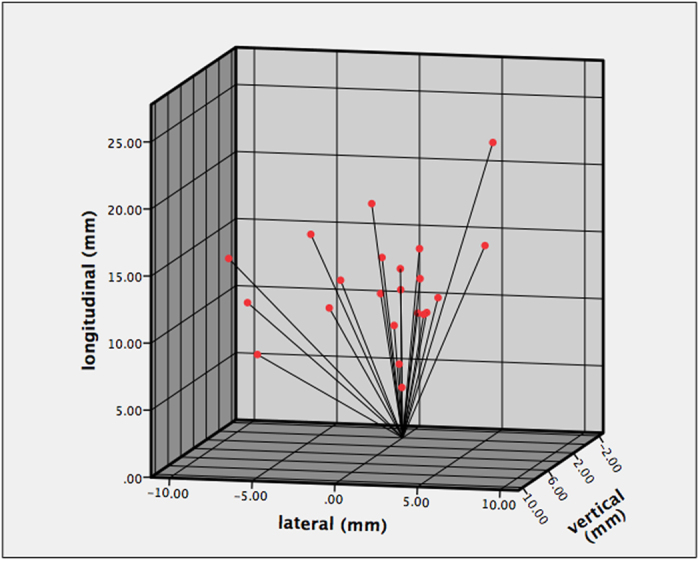
3D scatterplot of catheter spatial displacement between positions A and B.

Considering position A as the reference, significant displacement of the balloon center to position B was observed in the longitudinal direction. The average shift in the vertical, longitudinal and lateral directions was 2.91 mm (range: 0.10–9.00), 12.04 mm (range: 4.50–24.50) and 2.65 mm (range: 0.60–8.80), respectively. Figure 1 shows the spatial displacement in each patient, based on position A, which is defined as (0, 0, 0) in the 3D graph.

With respect to reproducibility, the average D_ICRU_ was 2.68 Gy (44.6%) in position A and 1.63 Gy (27.1%) in position B (*p* < 0.001). The D_ICRU_ ratio between positions A and B was 1.64. A significant decrease in maximum dose (*p* = 0.016) was also observed. For the other parameters, the position A/B ratio was 1.023 (D0.1cc), 1.006 (D1.0cc), 1.005 (D2.0cc), 1.032 (D4,0cc) and 1.027 (D50%). The percentage change in each parameter is listed in Table 2.

## Discussion

This study is the first controlled analysis that addresses the limitations of bladder point dosimetric data, in terms of reproducibility. Earlier reports on vaginal vault treatment have merely evaluated the differences between the averages values of ICRU bladder point dose and volumetric parameters; thus, this drawback of point dosimetry argues in favor of 3D planning.

Although the use of CT scan for dose calculation in VCB is attractive, no study has proven the superiority of volumetric data against classical ICRU points with regard to predicting urinary toxicity, necessitating a discussion of the cost-effectiveness of planning with tridimensional imaging in VCB compared with simple x-ray due to the minimal optimization of the applicators^13^,^14^,^15^, lack of clinical correspondence of the reported dose^16^ and significant rise in procedure costs^7^.

The ICRU 38 states that “the catheter is pulled downwards to bring the balloon against the urethra”^3^. This orientation is fundamental to the localization of the ICRU bladder point, since this document claims that “on the lateral radiograph, the reference point is obtained on an anterior-posterior line drawn through the center of the balloon. The reference point is taken on this line at the posterior surface of the balloon. On the frontal radiograph, the reference point is taken at the center of the balloon”. In this setting, due to the possible variation between different physicians, or even between different insertions performed by the same physician, our study evaluated the impact of various levels of tension (A-standard vs B-extra tension) on catheter spatial position and dose reported to the bladder.

The documented spatial change in the catheter exceeded 10 mm in 1 or more directions in 68% of the patients, translating into an overall reduction in the ICRU bladder point dose of 39% (44.6% to 27.1% of the prescribed dose) and confirming our hypothesis that an intrinsic limitation of the ICRU bladder point dosimetry in terms of reproducibility can lead to a bias. Additionally, Dmax, another point dose analysis, was also significantly affected by catheter position but to a lower extent (5% absolute reduction). Notably, the volume-based parameters were not influenced, with the exception of D50%. These findings could be explained by the hypothesis that the different tension level applied altered the ballon position (changing the ICRU measurement) but not the bladder anatomic position (determined by the constant volumetric doses) in relation to the high-dose region.

Two studies compared D_ICRU_ versus volumetric bladder dose that was reported by computed tomography planning using a single-lumen vaginal cylinder in the vaginal apex. Russo *et al*.^6^ published a retrospective dosimetric study, analyzing the Dmax and D2cc of the first 20 patients who were treated with CT-based 3D planning after a departmental policy change toward this method. They compared the doses to ICRU bladder point that was recorded from 71 patients who had been previously treated in the same institution, based on plain-film orthogonal radiographs. The average normalized bladder dose (as percentage of prescribed dose) was significantly higher with Dmax (x1.78) but not D2cc (x1.08), compared with D_ICRU_. These differences might be attributed to the lack of 2D and 3D information being collected from the same patients.

In the second study, Hung *et al*.^8^ prospectively examined the dosimetric effects of bladder filling on OARs and noted a reduction in D2cc values for the small bowel but not the bladder. As expected, the D50% (dose received by 50% of bladder volume) was significantly reduced with a full bladder, due to the increase in volume. Using a similar method as ours, after simulation of the ICRU point position on CT images, they recorded a mean D2cc/D_ICRU_ ratio of 0.95. The difference in mean D_ICRU_ between an empty and full bladder was 16 cGy and corresponded to an increase of 1.3% increase with an empty bladder (*p*-value not provided). These findings suggest the reproducibility of the ICRU point regarding bladder filling. Table 3 compares the characteristics of these studies.

In our series, the same physician delineated the OARs to limit the discrepancies in contouring between assistants. One possible limitation of our study was the absence of a CT scan in every insertion, because possible changes in bladder^8^,^9^,^10^ or rectal^11^ filling might have influenced the reported dose to the OARs. Moreover, Hoskin *et al*.^17^ documented that the angulation of the cylinder can also alter the dose in the bladder and rectum. It’s reasonable to hypothesize that the extra-tension could act as another source of displacement of vaginal vault, which was not controlled in our analysis, but should be tested in future studies. However, other studies suggest that the evaluation of the first insertion is sufficient in terms of dose reporting^9^,^18^; this is the current approach at our institution. Another limitation is the absence of a documented manometer reading, which can measure various levels of tension and help reproduce the findings of our study. The normalization that was performed by Russo *et al*.^6^, based on the prescribed dose as the denominator, is recommended for studies that compare different dose parameters and is essential for studies that use different doses per fraction, to make the results between studies comparable. In our data, the normalization was meant to account for differences in the prescription reference line, because patients who received VCB alone had 6 Gy/fraction prescribed to 0.5 cm from the cylinder surface, and those who received both VCB and EBRT were targeted at the surface.

Our group recently published a retrospective series on 126 patients who were treated with high-dose-rate VCB alone for intermediate-risk endometrial cancer with a 9.5% rate of grades 1 or 2 acute urinary toxicity and no case of grade 3 toxicity^16^. We did not find any differences in bladder ICRU point doses between the asymptomatic group and symptomatic group, with 11.256 Gy × 11.952 Gy (*p* = 0.69), respectively. Additionally, the use of a Foley catheter was deemed to be responsible for part of the acute toxicity reported, due to an inherent increased risk of urinary tract infection^7^,^16^. Despite the low rates of grades 3 and 4 urinary toxicity in the literature after VCB alone (<3%)^5^, the combination with EBRT could lead to serious complications, like bowel perforation and urethral strictures^1^. Additionally, the documentation of the previous treatment dose to OARs can more accurately define an opportunity for re-irradiation, in cases of recurrence.

Nevertheless, contrasting results have been reported by studies that have addressed the dosimetric difference of point versus dosimetric parameters in high-dose-rate brachytherapy for radical treatment of cervical cancer (intact uterus). Some studies suggest a tendency in the correlation^19^,^20^, whereas others do not^21^. Although our findings support the adoption of CT-based planning in VCB, its generalization to HDR in the intact uterus should be supported by specific studies.

Further, additional studies are necessary to identify better predictors of toxicity in HDR brachytherapy for the vaginal vault and for radical treatment of cervical cancer and inoperable endometrial cancer. In these settings, our findings might support future trial designs that include 3D planning. It might be better to define the extension or particular anatomy of the vaginal vault and superior vaginal mucosa, leading to changes in the prescription method to volumetric instead of merely using a reference from the applicator surface or target surrogates.

In conclusion, the dose reported to the bladder using bladder D_ICRU_ or Dmax is dependent on the tension of the balloon in VCB. Because volumetric parameters (D0.1cc, D1.0cc and D2.0cc) are independent of the anatomical changes due to the presence of a urinary catheter, they might be a better option. Further analysis of toxicity outcomes is needed to determine the most appropriate dose parameter.

## Methods

### Patients

The study recruited female patients aged between 18 and 85 years. The inclusion criteria were a histological diagnosis of cervical or endometrial cancer and a computed tomography scan (CT scan) that was available in the planning system. Patients who did not undergo oncological surgery as the initial treatment were excluded. The institutional review board, named *Comitê de Ética em Pesquisa em Seres Humanos da Fundação Antônio Prudente*, approved this study design and the use of patient information without name or facial identification. All patients signed informed consent forms before study entry. The protocol was registered earlier (NCT02091050), and the methods were carried out in accordance with the approved guidelines.

### Simulation and Insertions

**Figure 2:**
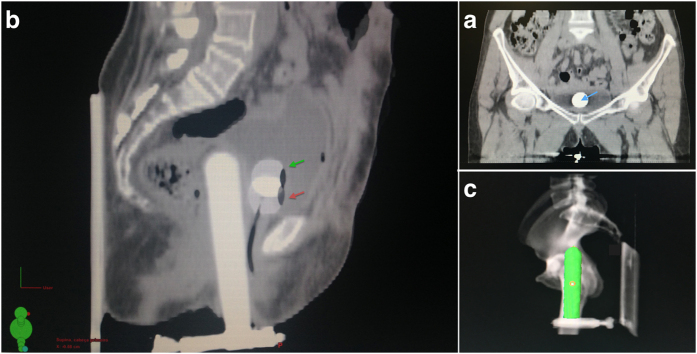
(**a**) Sagittal view of the fused planning CT scan at both tension levels: standard (position A, green arrow) and extra tension (position B, red arrow). (**b**) Bladder reference point identified at the center of the balloon on a coronal plane (blue arrow). (**c**) Digitally reconstructed radiograph (DRR) from the CT scan in position A.

Before insertion of the applicator, a Foley catheter was inserted into the urethra, to report the ICRU bladder dose (D_ICRU_), as described in the ICRU 38 report^3^, but adapted to the computed tomographic image. Briefly, on the sagittal view of the tomographic image, the bladder reference point was obtained on the posterior surface of the balloon, on an anterior-posterior line through the center of the balloon. On the coronal view, the reference point was defined as the center of the balloon, mimicking the position in the traditional simple x-ray image (Fig. 2a).

To test the robustness of the dose parameters, CT scan images were obtained in two scenarios: with the Foley catheter tensioned towards the caudal direction of the patient using standard tension and extra tension (Fig. 2b). Standard tension (position A) was defined as the tension that was necessary to position the balloon at the bladder trigone. Extra tension (position B) was defined as additional tension in the catheter, limited by the patient’s complaint of discomfort. The CT scan had the following characteristics: voltage 120 kV, current 275 mA and exposure 300 mAs. A manometer was not used to identify different tension levels. After insertion of the vaginal applicator, limited effort was made to correct the insertion angle to a central position in the pelvis before performing the CT-scan. It was not provided a specific instruction concerning bladder filling. Because the two images were taken in a short interval (less than 2 minutes), the volume of the bladder was considered to be constant between positions A and B (average 350.560.4 mL and 360.4 mL, respectively).

Lateral and anteroposterior simple x-ray images were taken before each subsequent insertion to confirm the position of the cylinder, comparing the radiographic image with a digitally reconstructed radiograph (DRR) from the CT-scan in position A (Fig. 2c). Again, limited effort was made to correct the insertion angle, with posterior documentation of the final position with the other set of x-ray images. The lithotomy position was used only during insertion of the applicator, and the patients underwent the simulation and treatment with their legs extended.

### Prescription and Dosimetry

The total dose was 24 Gy, delivered in 4 weekly fractions of 6 Gy (EQD2 38.4 Gy) to the cylinder surface in EBRT + VCB cases and 0.5 cm from the applicator surface in cases that were treated with VCB alone, to cover more of the mucosa. Because no patient presented with vaginal extension of the tumor, based on the pathological report, the cylinder was activated only at the proximal 2 cm, at the department’s discretion.

The brachytherapy planning and dosimetric data were obtained from the treatment planning system (BrachyVision Eclipse®, version 11.0, Varian, Palo Alto, CA, USA), based on the CT-scan that was performed before the first procedure. The same physician contoured the OARs in all images. The dose parameters analyzed were: bladder reference point (D_ICRU_); maximum bladder point (Dmax bladder); the minimum dose value in the 0.1 cc, 1.0 cc, 2.0 cc and 4.0 cc receiving the highest dose (D0.1cc, D1.0cc, D2.0cc, D4.0cc) and the dose that was received by 50% of the bladder (D50%).

The treatment was delivered using a GammaMedplus™ iX high-dose-rate device (VARIAN, Palo Alto, CA, USA), using the AAPM TG-43 formalism. The ^192^Ir Gammamed HDR plus source listed in the library of origin was used.

### Statistical analysis

Visual observation of the histograms and Shapiro-Wilk’s test^22^ (p > 0.05) were used to define the normality of the distribution of doses. Wilcoxon signed rank test^23^ was used to compare doses of non-normally distributed dependent variables. All statistical analyses were performed using IBM SPSS Statistics, version 20.0. Armonk, NY.

## Additional Information

**How to cite this article**: Sapienza, L. G. *et al*. Volumetric (3D) bladder dose parameters are more reproducible than point (2D) dose parameters in vaginal vault high-dose-rate brachytherapy. *Sci. Rep*. **6**, 28074; doi: 10.1038/srep28074 (2016).

## Figures and Tables

**Table 1:** Comparison between ICRU bladder dose point and other bladder dose parameters.

With standard tension		With extra tension	
D_ICRU_ × Dmax	**p** **<** **0.001**	D_ICRU_ × Dmax	**p** **<** **0.001**
D_ICRU_ × D0.1cc	**p** **<** **0.001**	D_ICRU_ × D0.1cc	**p** **<** **0.001**
D_ICRU_ × D1.0cc	**p** **=** **0.001**	D_ICRU_ × D1.0cc	**p < 0.001**
D_ICRU_ × D2.0cc	**p = 0.003**	D_ICRU_ × D2.0cc	**p < 0.001**
D_ICRU_ × D4.0cc	**p = 0.020**	D_ICRU_ × D4.0cc	**p < 0.001**
D_ICRU_ × D50%	**p < 0.001**	D_ICRU_ × D50%	**p < 0.001**

**Table 2:** Summary of statistics by parameter and tension applied to Foley catheter.

Parameters	Foley catheter tension (normalized*)		% change	*p* value
	Standard tension	Extra tension		
D_ICRU_	44.6	27.1	39.2	**p < 0.001**
Dmax	103.5	98.3	5.0	**p = 0.016**
D0.1cc	88.1	86.1	2.3	p = 0.123
D1.0cc	72.3	71.8	0.7	p = 0.390
D2.0cc	65.3	65	0.5	p = 0.372
D4.0cc	57.1	57	0.2	p = 0.269
D50%	12.3	12	2.4	**p = 0.002**

^*^Normalized as percentage of prescribed dose. SD = standard deviation. D_ICRU_ = dose reported using ICRU bladder point visualized in CT plan.

**Table 3:** Design of published studies that have compared bladder D_ICRU_ and other parameters in VCB.

Study	Year	n	Design	CT planning	Ratio Dmax/D_ICRU_	Ratio D2cc/D_ICRU_
Russo *et al*.^6^	2012	71 (2D) 20 (3D)	retrospective	every fraction	1.79	1.08
Hung *et al*.^8^	2012	12 (2D and 3D) empty bladder	prospective	1st fraction only	NR	0.92
		12 (2D and 3D) full bladder			NR	0.98
present study	2015	22 (2D and 3D) standard tension	prospective	1st fraction only	2.32	1.46
		22 (2D and 3D) extra tension			3.62	2.39
